# Zebrafish xenograft model for studying mechanism and treatment of non-small cell lung cancer brain metastasis

**DOI:** 10.1186/s13046-021-02173-5

**Published:** 2021-11-20

**Authors:** Ruo-Yue Fan, Jia-Qi Wu, Yu-Yang Liu, Xiang-Yu Liu, Si-Tong Qian, Chong-Yong Li, Ping Wei, Zhe Song, Ming-Fang He

**Affiliations:** 1 College of Biotechnology and Pharmaceutical Engineering, Nanjing Tech University, 30 Puzhu South Road, 211816 Nanjing, P. R. China; 2 Jiangsu Tripod Preclinical Research Laboratory Co. Ltd, 211816 Nanjing, China; 3 Department of Neurosurgery, The Affiliated Drum Tower Hospital, School of Medicine, Nanjing University, 210023 Nanjing, China; 4 Key Laboratory of Drug Quality Control and Pharmacovigilance, Ministry of Education, China Pharmaceutical University, 210009 Nanjing, China

**Keywords:** Zebrafish, Brain metastasis, Xenograft, Non-small cell lung cancer

## Abstract

**Background:**

Brain metastasis (BM) is thought to be related to the mortality and poor prognosis of non-small cell lung cancer (NSCLC). Despite promising development of NSCLC treatment, the treatment of NSCLC BM is still not optimistic due to the existence of the blood-brain barrier (BBB) that prevent drug penetration, as well as the short median survival time of the patients left for treatment. In this context, further development of quick and effective pre-clinical models is needed in NSCLC BM treatment. Here, we report a model system using zebrafish to promote the development of drugs for patients with NSCLC BM.

**Methods:**

Three different NSCLC cell lines (H1975, A549 and H1299) were used to establish zebrafish BM models. The embryo age and cell number for injection were first optimized. Metastatic cells were observed in the brain blood vessels of zebrafish and were verified by hematoxylin-eosin (HE) staining. Then, the metastasis potentials of H1975 and A549 with manipulated microRNA-330-3p (miR-330-3p) expression were also investigated. Finally, sensitivities of H1975 and A549 to osimertinib and gefitinib were tested.

**Results:**

This zebrafish BM model could distinguish NSCLC cell lines with different BM potential. Over-expressed miR-330-p significantly improved the BM potential of the A549 cells while knockdown miR-330-p reduced the BM ability of the H1975 cells. Both osimertinib and gefitinib showed inhibition effect in zebrafish BM model with the inhibition rate higher than 50 %. H1975 cell showed much higher sensitivity to osimertinib rather than gefitinib both *in vivo* and *in vitro*.

**Conclusions:**

We established zebrafish brain metastasis model for studying mechanism and treatment of NSCLC BM. This study provided a useful model for NSCLC brain metastasis that could be used to study the mechanism that drive NSCLC cells to the brain as well as identify potential therapeutic options.

**Supplementary Information:**

The online version contains supplementary material available at 10.1186/s13046-021-02173-5.

## Background

Although the development of targeted treatments and biomarkers is promising, non-small cell lung cancer (NSCLC) is still one of the leading cause of cancer-related mortality [1]. Most NSCLC-associated deaths result from metastases that are resistant to conventional therapies (surgical resection, radiotherapy, chemotherapy or a combination of these) [2], the most common of metastasis is brain metastasis (BM). 30–43 % of patients with NSCLC develop BM alone with no evidence of metastatic disease elsewhere [3], and 30 %-54 % of NSCLC patients will develop BM after treatment [4]. The median survival time of NSCLC BM patients is 7-8 months [5]. BM is also associated with poor prognosis and portend limited effective treatment options [6].

For NSCLC patients with multiple BM, whole brain radiotherapy is still the main method [7]. Although whole brain radiotherapy has an irreplaceable status in BM currently, its toxic effects should warrant enough attention, such as cognitive decline and symptomatic radiation necrosis [8, 9]. Chemotherapy is not so often an effective approach for NSCLC BM, due to its low blood-brain barrier (BBB) permeability and side effects. In the treatment of NSCLC, successful application of EGFR and anaplastic lymphoma kinase (ALK)/met proto-oncogene tyrosine kinase inhibitors highlights the importance of genotype-based individualized targeted therapies [10–12]. However, due to the extensive molecular and functional heterogeneity, BM patients benefit limited from targeted therapy. On the one hand, not all targeted drugs have good BBB permeability and the singularity of the targets limits its effect in the BM with multiple mutations [13] On the other hand, although some targeted drugs with good BBB penetration, such as osimertinib, show good activity in BM patients in the early stage of treatment [14], acquired resistance to mutant EGFR (T790M) can evolve following osimertinib treatment [15]. Besides, despite the encouraging data, only few patients respond to immunotherapy [16]. In general, no matter what kind of treatment methods, there are huge individual differences in the efficacy of NSCLC BM patients, which urgently need a preclinical animal model to assist precise medication.

Zebrafish (*Danio rerio*) is a powerful and genetically manageable model that can be used to study human malignancies. It shows a high degree of physiological and genetic similarity to mammals, closely mimics the clinical environment and allows monitoring of the natural course of the tumor [17, 18]. Besides, zebrafish embryos are transparent, allowing dynamic *in vivo* observation of cancer cell proliferation, invasion and metastasis [17, 19]. Zebrafish xenograft models are used in a variety of cancer research. In gastric cancer research, the zebrafish patient-derived xenograft model showed clinically similar pathological phenotypes and drug sensitivity [20]. It is also reported that zebrafish xenografts are a fast and highly sensitive assay that can be used to display multiple biological tumor traits and assess tumor response to treatment of colorectal cancer [21]. Due to the high similarity of its BBB structure and function to humans, the zebrafish orthotopic glioblastoma xenograft models are used to screen blood-brain barrier penetrating drugs [22, 23]. Although there are many zebrafish models used for cancer metastasis research [24–27], there is a lack of zebrafish xenograft models to study brain metastasis, especially NSCLC brain metastasis.

Herein, we used different NSCLC cell lines (H1975, A549 and H1299) to establish zebrafish BM models, and verified its feasibility and reliability in the mechanism research and treatment of NSCLC BM. We demonstrated that this zebrafish NSCLC BM model could simulate human NSCLC BM. It could be a useful model to study the mechanisms that drive NSCLC cells to brain as well as identify potential therapeutic options.

## Methods

### Reagents

Osimertinib (Osi, purity > 99 %) and gefitinib (Gefi, purity > 99 %), purchased from Selleck Chemicals (Shanghai, China), was dissolved with DMSO to obtain a stock concentration of 100 mM and 20 mM. Diluted solutions were made before experiments were performed. 0.1 % DMSO in embryo medium or cell culture medium was used as solvent control. Fetal bovine serum (FBS), phosphate buffer saline (PBS), Roswell Park Memorial Institute basal medium 1640 (RPMI 1640), penicillin and streptomycin were purchased from Basal Media Technologies (Shanghai, China). Matrigel matrix, purchased from Corning, was used at a one-to-eight dilution.

### Cell lines and culture

Human NSCLC lung cancer cell lines, A549, H1975, H1299, originally from American Type Culture Collection, were cultured in accordance with standard requirements. Human breast cancer cell lines, MDA-MB-231 and MCF-7 was purchased from Procell (Wuhan, China). A549 was cultured in DMEM supplemented with 10 % FBS and 1 % Penicillin-Streptomycin. H1299 and H1975 were cultured in RPMI 1640 supplemented with 10 % FBS and 1 % Penicillin-Streptomycin. MDA-MB-231 was cultured in L15 supplemented with 10 % FBS and 1 % Penicillin-Streptomycin without CO2. MCF-7 was cultured in MEM supplemented with 10 % FBS, 0.01 mg/mL insulin and 1 % Penicillin-Streptomycin. All cells were cultured in a humidified atmosphere containing 5 % CO2 at 37℃.

### Zebrafish lines and maintenance

Zebrafish and embryos were raised in accordance with standard procedures. Adult fish undergo a light-dark circle of 14 h (h) /10 hours (h). Adult fish aged 3 months to 2 years were crossed to produce embryos and larvae. Tg (*fli-1*: EGFP), allowing the visualization of the vascular systems in zebrafish, and wild-type AB zebrafish were used in this study. Embryos and larvae were cultured in E3 medium (5 mM NaCl, 0.17 mM KCl, 0.33 mM CaCl_2_, 0.33 mM MgSO_4_) at 28℃, 0.2 mM N-phenylthiourea (PTU; Sigma) was applied to prevent pigment formation from 1 day post-fertilization (dpf) in order not to interfere with imaging. The zebrafish studies were approved by the Institutional Animal Care and Use Committee (IACUC) at Nanjing Tech University.

### *In vitro* cell invasion ability assay

Transwell culture system (Cell Culture Inserts, 8 μm, 24-well; Corning, China) was used to assess cell invasion ability. Spread the diluted Matrigel matrix with serum-free medium in the chambers and put them in the 37 °C cell incubator to make them solidify. The cells pre-starved with serum-free medium for 24 h were configured to a certain density, then 200 µL of the cell suspension were added to the upper chamber, 500 µL of complete medium were added to the lower chamber, and the plate was put in cell incubator for 24 h. Cells at the bottom of the lower chamber were fixed with neutral formaldehyde for 40 min (min) and washed with PBS twice. Put the chamber in 0.5 % crystal violet solution for 30 min to label the cells, then picture them by microscope and count them using Image Pro Plus.

### Cell labelling

Cell lines were labelled with CM-DiI (Thermo Fisher Scientific, Waltham, MA, USA) at a concentration of 0.5 µL/mL, and CM-DiO (Yifeixue Bio Tech, China) at a concentration of 8 µL/mL according to manufacturer’s instructions.

### Cell Transfection

After determining the appropriate transfection concentration, the cells were starved for 24 h, then cultured with the transfection reagent for 24 h, according to manufacturer’s instructions. Transfected cells were verified by qPCR. The sequence of miR-330-3p mimics was GCAAAGCACACGGCCUGCAGAGAUCUGCAGGCCGUGUGCUUUGCUU and the sequence of miR-330-3p inhibitor was UCUCUGCAGGCCGUGUGCUUUGC. All RNA oligos were provided by GenePharma (China).

### Zebrafish xenograft

Zebrafish embryos at indicated developmental stages in each experiment were anaesthetized with 0.003 % tricaine (Sigma-Aldrich, St. Louris, MO, USA) and placed on a 10 cm Petri dish coated with 1 % agarose. The single cell suspension, formulated into certain density of 2 × 10^7^ cells per milliliter, was injected into perivitelline space by microinjection. Each embryo beard about 100 cells to cause brain metastasis. Suitable injected embryos were selected after 2 h post injection (hpi). Selected embryos were placed in 32℃ for subsequent experiments according to our previous reports [20].

### Zebrafish xenograft drug administration

The 1-day-post-injection (dpi) zebrafish xenografts with similar size of brain metastasis were randomly distributed in the treatment group: control E3 medium, Osimertinib in E3 (1 µM) and Gefitinib in E3 (13 µM) for three consecutive days. All drug administration were by intracardiac injection.

### Quantitative Real-time PCR (qPCR)

The brain tissues were isolated from embryos and collected, and RNA was extracted from them using an RNA extraction kit (Yifeixue Bio Tech) according to manufacturer’s instructions. Using Quantitative Real-time PCR to quantity miR-330-3p (Forward: GCCAACAATATCCTGGTGCTG; Reverse: GAGGTATTCGCACTGGATACGACTCTCTG), *mfsd2aa* (Forward: CTCTTCACTTCGCTAGCCTTCATG; Reverse: CGATGTAAACAGCAGTCTTTTTCCC), *mfsd2ab* (Forward: TCTCGACTCTTAGTCTTGATTTCGC; Reverse: GAGTCCGTTTCTGAATCCATCTCG), *Claudin-5* (Forward: GCCCACTAAAAGAGCCACAT; Reverse: AGAGTCCAGCGAAAAGCATC).

### Hematoxylin-eosin (HE) staining

At 4 dpi, embryos with NSCLC brain metastasis were fixed in 4 % paraformaldehyde, then dehydrated, paraffin embedded and sectioned (6–8 μm). Sections were stained with HE. The images were acquired with biopathology microscope (BX45-DP72, Olympus, Japan).

### Imaging

Tumor cells migration in brain were monitored at 1 dpi and 4 dpi by an inverted fluorescence microscope (IX71, Olympus, Japan) or confocal microscope (LSM710, ZEISS, Germany).

### Statistical analysis

All statistical analyses were performed using Image-Pro Plus 6.0 and GraphPad Prism 8.0. All statistical analyses were expressed as mean ± SEM. The decrease/increase in fold of change was analyzed using one-way ANOVA followed by Dunnett multiple comparison test. Significance was considered when P values were lower than 0.05. (***) indicates statistical significance P < 0.005, (**) P < 0.01, (*) P < 0.05. All experiments were done in triplicates and independent experiment was repeated at least three times.

## Results

### Establishment of zebrafish NSCLC BM xenograft models

**Fig. 1:**
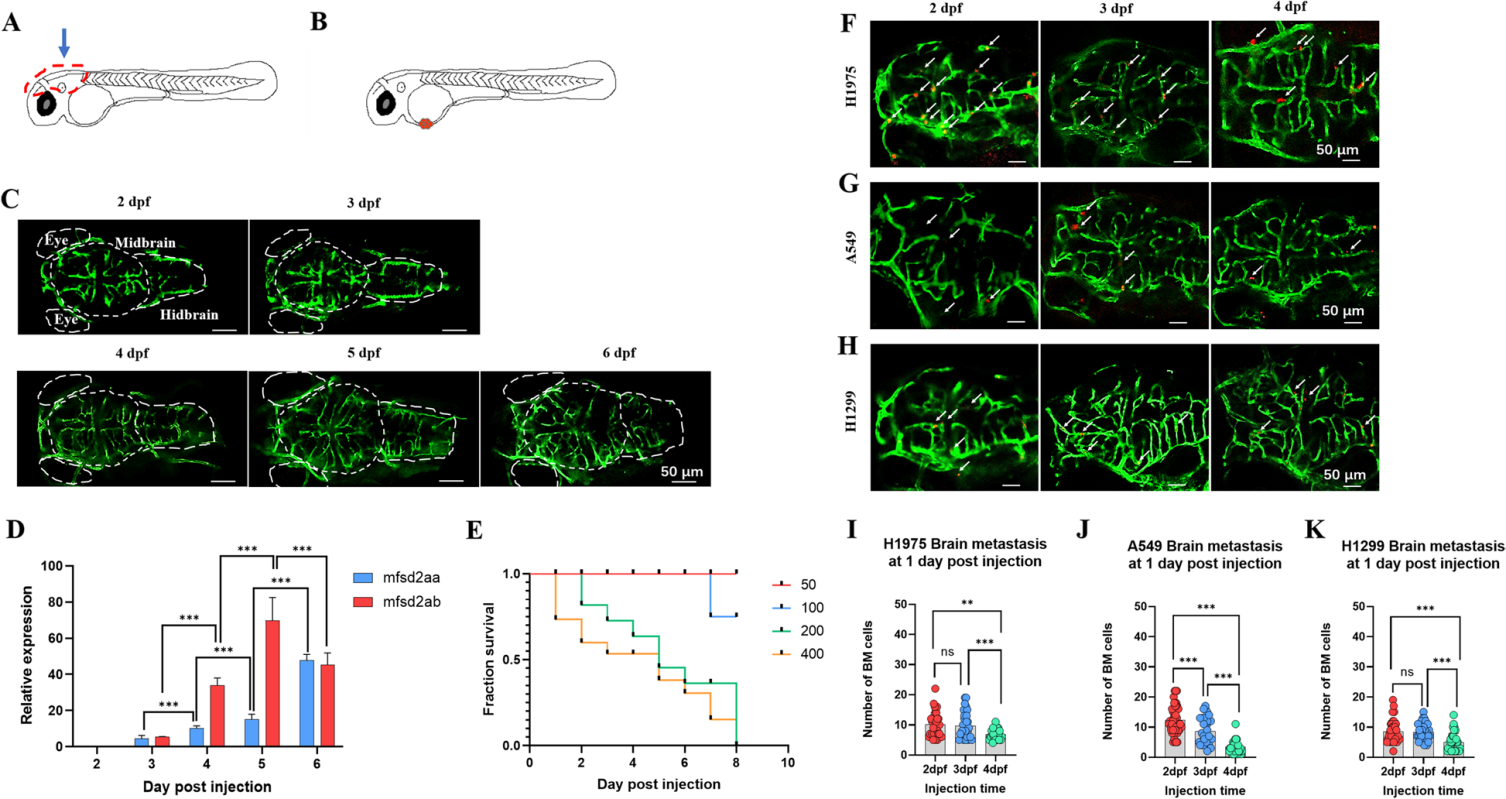
Establishment of zebrafish NSCLC BM xenograft models. (**A**) Schematic diagram of imaging and collecting zebrafish brain. The zebrafish brain was imaged from vertical view (blue arrow) and excised (red dotted box) to extract RNA. (**B**) The schematic diagram showed zebrafish embryos at 2 dpf. The red dots indicated cancer cells injected into the perivitelline space (PVs). (**C**) Images of brain at different developmental stages of Tg (*fli-1*: EGFP) zebrafish (2-6 dpf). The white short dashed line indicated the midbrain of the zebrafish, and the white long dashed line indicated the zebrafish’s eyes and hindbrain. (**D**) The expression of *claudin-5* in the zebrafish brain at different developmental stages (2-7 dpf). (**E**) The expression of *mfsd2aa* and *mfsd2ab* in the zebrafish brain at different developmental stages (2-6 dpf). (**F**) Survival curves of 2 dpf zebrafish with different tumor-bearing amount in PVs. About 50-400 H1975 cells (red fluorescence) were injected into the PVs of 2 dpf zebrafish, and the number of deaths was counted till 8 dpi. (**G**-**I**) Three human NSCLC cell lines were involved: H1975, A549 and H1299. About 100 cells were injected into the PVs of zebrafish at different developmental stages, and the brain of zebrafish was imaged at 1 dpi. The white arrows indicated cancer cells in blood vessels of zebrafish brain. (**J**-**K**) Quantification of the BM cells number in zebrafish at 1 dpi. Significance was considered when P values were lower than 0.05. (ns) indicated statistical insignificance, (*) indicated statistical significance P < 0.05, (**) P < 0.01 and (***) P < 0.001. dpf: days post fertilization, dpi: days post injection

BM of NSCLC is associated with the brain microenvironment [28], so we first evaluated the structure and function of the zebrafish BBB and its impact to the BM. As the schematic diagram shown, the red dotted box indicated the brain of zebrafish (Fig. 1 A). Tg (*fli-1*: EGFP) zebrafish was used to observe the development of blood vessels in brain at different developmental stages (2-6 dpf). The structure of zebrafish BBB was mainly composed of blood vessels in brain [29]. We observed that the density of blood vessels in brain significantly increased at 4 dpf (Fig. 1 C). The expression of *claudin-5*, a tight junction marker of blood vessels in the BBB, gradually increased with brain development, especially there was a significant increase of expression between 4 dpf and 3 dpf (Fig. 1D). Zebrafish contain two paralogues of *mfsd2aa* and *mfsd2ab*, both of them express in the developing zebrafish central nervous system. The expression of *mfsd2aa* was used to mark the beginning of BBB maturation and kept increasing until 3 months post fertilization. The first expression of *mfsd2ab* meant that the midbrain of zebrafish had mature functional BBB, and the decrease of expression meant the end of functional development [29, 30]. We next investigated the expression of *mfsd2aa* and *mfsd2ab* in zebrafish brains at different developmental stages (2-6 dpf). *Mfsd2aa* and *mfsd2ab*, almost no expression at 2 dpf, began to express at 3 dpf and significantly increased at 4 dpf. The expression of *mfsd2ab* reached its peak at 5 dpf and decreased significantly at 6 dpf. The expression of *mfsd2aa* continued to increase at 5 dpf and 6 dpf (Fig. 1E). These results showed that the BBB of zebrafish may began to functional develop at 3 dpf, basically developed at 4 dpf, and fully developed at 5 dpf. Therefore, three time points were selected—2 dpf (the BBB was not developed), 3 dpf (the BBB was developing) and 4 dpf (the BBB was basically developed)—to evaluate whether the existence of BBB affected the BM potential of NSCLC cell lines.

To establish the zebrafish xenograft BM model, optimal injection cell number was first determined by making survival curves of 2 dpf zebrafish embryos xenografted with different amounts of cancer cells. A series of H1975 cells (50, 100, 200, 400 cells/embryo) were injected into the PVs of 2 dpf zebrafish, then the survival rate was calculated till 8 dpi. As indicated in Fig. 1 F, when the number of injection cells were 50 or 100, it had little effect on the survival of zebrafish till 6 dpi. Since the injection number of 50 cells could not cause significant BM (data not shown), we chose 100 as the number of injection cells to accurately observe and quantify the BM of zebrafish.

Next, the optimal injection time was determined. Three different NSCLC cell lines were involved: H1975, A549 and H1299. Cancer cells were labelled with CM-DiI, a red fluorescent dye, and injected into the PVs of zebrafish embryos at different developmental stages (2, 3, 4 dpf). The zebrafish midbrains were imaged under fluorescent microscope (Fig. 1G and I) and the number of BM cells was quantified at 1 dpi (Fig. 1 J- 1 M). As indicated in Fig. 1G J, the BM potential of H1975 injected at 2 dpf was significantly higher than that injected at 4 dpf, which meant the BM potential of H1975 seemed to be weaken by the existence of developed BBB. This could also be reflected in A549 (Fig. 1 H and 1 K) and H1299 (Fig. 1I M). Despite the existence of developed BBB weakened the BM potential of NSCLC cell lines, it could not completely prevent brain metastasis of NSCLC cell lines—BM cells could be seen clearly in brain of zebrafish even if the cells were injected at 4 dpf (Fig. 1G and I). In order to better quantify the brain metastasis of NSCLC cell lines in zebrafish, 2 dpf was chosen as the injection time to construct zebrafish BM models, and used for subsequent verification.

### Hematoxylin-eosin (HE) stanning of zebrafish with NSCLC BM at 4 dpi

**Fig. 2:**
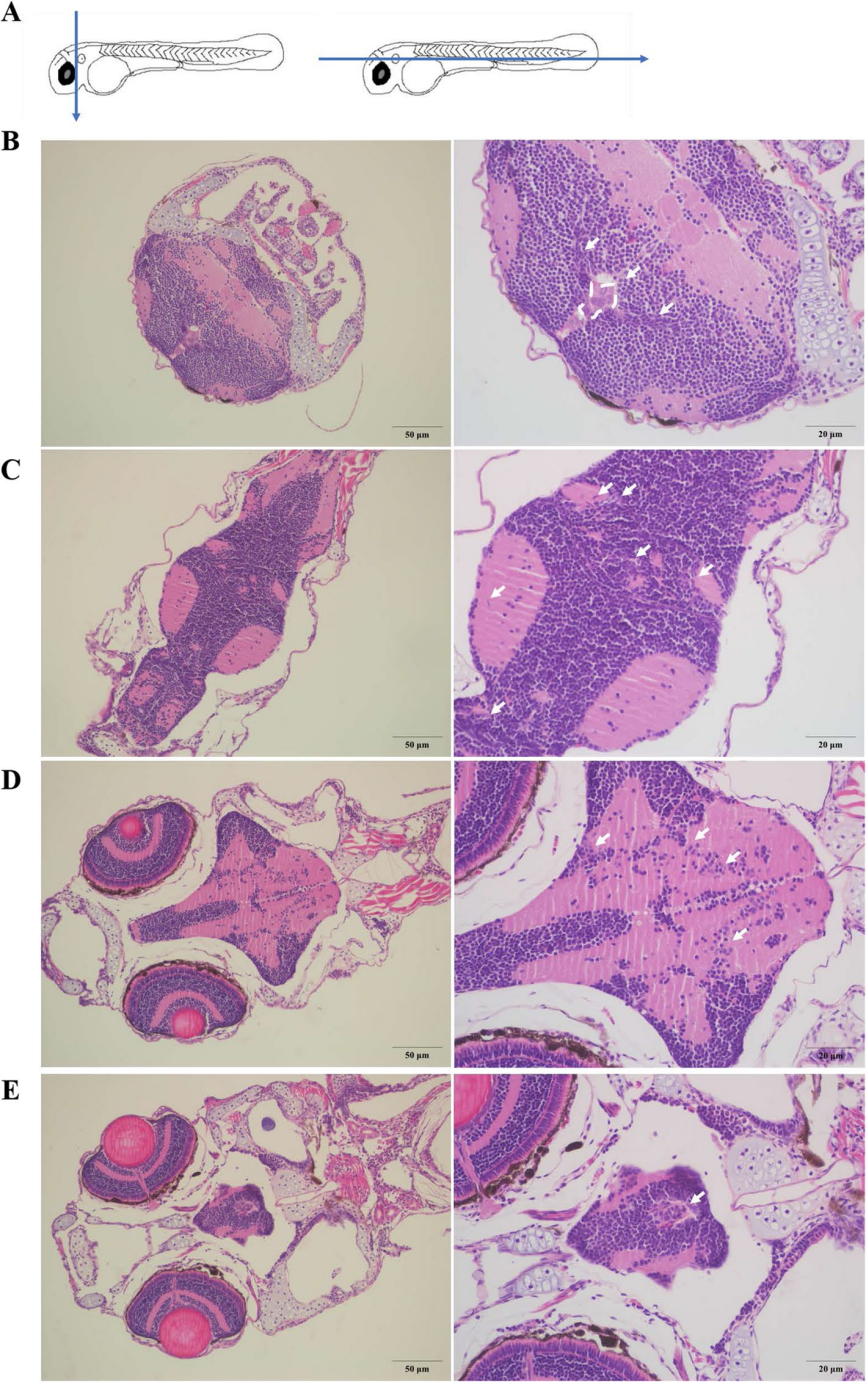
Hematoxylin-eosin (HE) stanning of zebrafish with NSCLC BM at 4 dpi. (**A**) Zebrafish embryos with NSCLC BM were sliced according to the trajectory (blue straight line) at 4 dpi. (**B**) The vertical sections showed that there were obvious cancer cell clusters (white dotted line) and lesions (white arrows) in the brain of zebrafish. (**C**-**E**) The picture showed three horizontal slices of the same zebrafish brain, from the surface layer to the inner layer. The slices showed that there were cancer cells (white arrows) in different levels of the zebrafish brain

About 100 H1975 cells were injected into the PVs of 2 dpf zebrafish, and the embryos at 4 dpi with BM were fixed and sliced according to the trajectory (blue straight line) and stained with HE (Fig. 2 A). Hematoxylin-eosin (HE) staining mainly distinguishes normal and abnormal cells by the morphology and size of the nucleus [31]. H1975 cells could be found in the brain of zebrafish at almost single cell resolution (white arrows). These individual cancer cells were long and narrow and had a similar morphology to H1975 cells cultured *in vitro*. In addition, the clustered nuclei were large and uneven in shape (white dotted frame), while the nucleus of normal cells is regular and uniform. The vertical sections showed that there were obvious cancer cell clusters (white dotted frame) and lesions (white arrows) in the brain of zebrafish (Fig. 2B). Three horizontal slices of the same zebrafish brain, from the surface layer to the inner layer were showed, H1975 cells could be found in all layers (white arrows) (Fig. 2 C-E). HE staining results indicated that NSCLC cancer cells could metastasized to multiple layers in the brain of zebrafish, which were correlate to the above study (Fig. 1G and J).

### NSCLC cells could not proliferate in the midbrain of zebrafish at 4 dpi

**Fig. 3:**
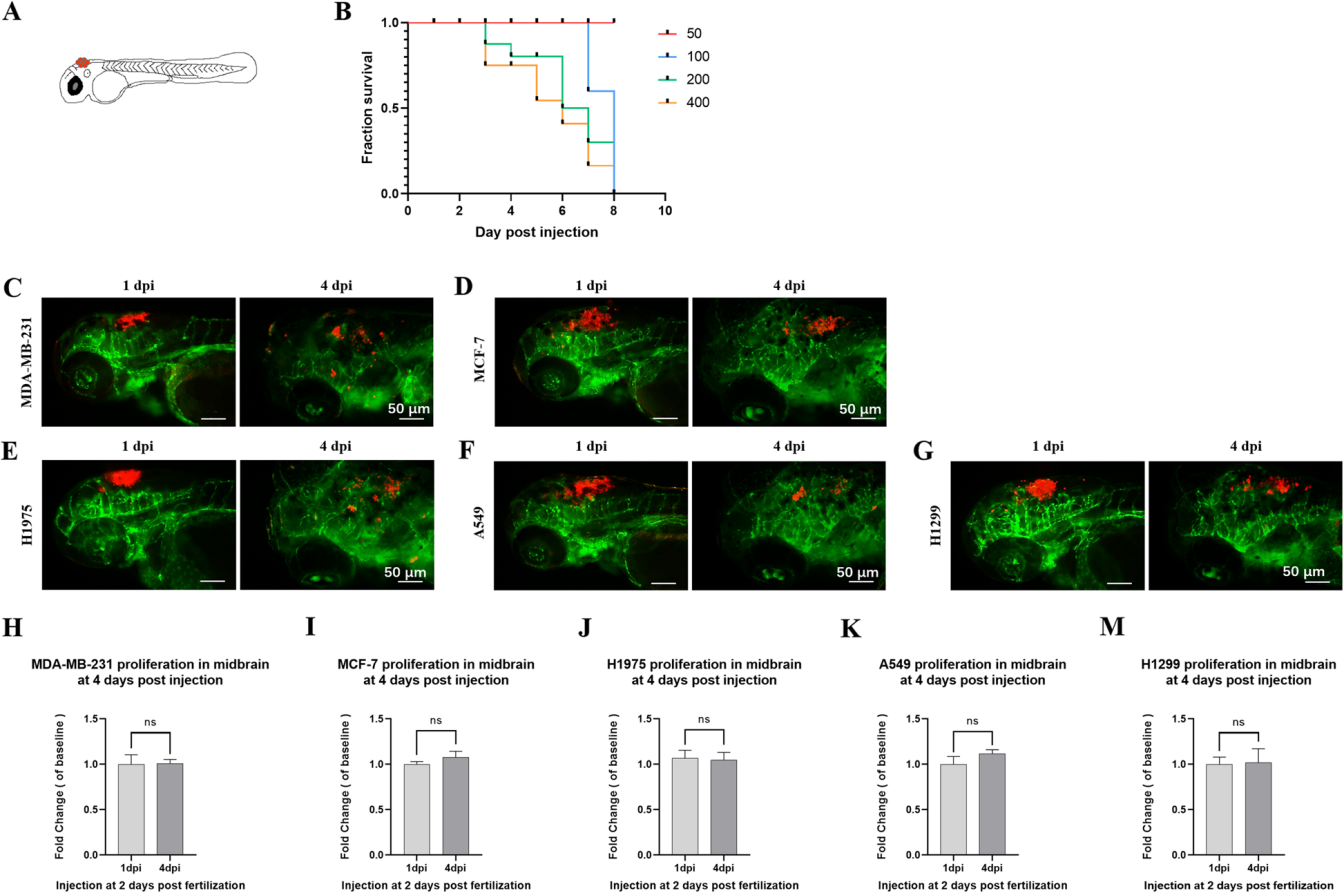
NSCLC cells could not proliferate in the midbrain of zebrafish at 4 dpi. (**A**) The schematic diagram showed zebrafish embryos at 2 dpf. The red dots indicated cancer cells injected into the midbrain of zebrafish. (**B**) Survival curves of zebrafish with different tumor-bearing amount at midbrain. About 50-400 H1975 cells (red fluorescence) were injected into the midbrain of 2 dpf Tg (*fli-1*: EGFP) zebrafish, and the number of deaths was counted till 8 dpi. (**C**-**G**) Five human cancer cell lines were involved: human breast cancer cell lines MDA-MB-231 (positive control) and MCF-7 (negative control), human NSCLC cancer lines H1975, A549 and H1299. About 100 cells were injected into the midbrain of zebrafish at 2 dpf, and the midbrain of zebrafish was imaged at 1 dpi and 4 dpi. (H-M) Quantification of cell proliferation in the midbrain of zebrafish at 1 dpi and 4 dpi. The fold change of cells in the midbrain was determined by dividing the measured values at 1 dpi and 4 dpi by the average measured values at 1 dpi. (ns) indicated statistical insignificance, (*) indicated statistical significance P < 0.05, (**) P < 0.01 and (***) P < 0.001

In above study, NSCLC cancer cells injected into the PVs of 2 dpf zebrafish were found to increase in the brain of zebrafish at 4 dpi (Fig. 1G-I). However, we could not determine if the observed cancer cells came from several cells that managed to extravasate or clonal expansion of just a few that managed to extravasate, i.e., cell extravasation vs. cell proliferation. Next, cancer cells were injected into the midbrain of 2 dpf Tg (*fli-1*: EGFP) zebrafish to evaluate whether the cancer cells could proliferate at 4 dpi (Fig. 3 A). Optimal injection cell number was determined by making survival curves of 2 dpf zebrafish with different tumor-bearing amount in midbrain. About 50-400 H1975 cells (red fluorescence) were injected into the midbrain of 2 dpf zebrafish, and the number of deaths was counted till 8 dpi. As indicated in Fig. 3B, when the number of injected cells was 50 or 100, it had little effect on the survival of zebrafish till 6 dpi. Considering that 50 NSCLC cells could not proliferate effectively in yolk sac of 2 dpf zebrafish (data not shown), 100 cells were selected as the injection cell number.

Five human cancer cell lines were involved: MDA-MB-231 (positive control), MCF-7 (negative control), H1975, A549 and H1299. Human breast cancer cell line MDA-MB-231 was reported as a highly aggressive cancer cell line, which could metastasize and proliferate in zebrafish [32]. Human breast cancer cell line MCF-7 was reported as a low invasive cancer cell line, compared to MDA-MB-231 [33]. Therefore, MDA-MB-231 was selected as positive control, and MCF-7 was selected as negative control. About 100 cells were injected into the midbrain of zebrafish at 2 dpf. The midbrain of zebrafish was imaged (Fig. 3 C-G) and the cells in midbrain were quantified at 1 dpi and 4 dpi (Fig. 3 H-M). Despite the data showed that all these cell lines did not proliferate in the midbrain of zebrafish, MDA-MB-231, H1975, A549 and H1299 showed a tendency to spread at 4 dpi (Fig. 3 C and E-G). On the other hand, MCF-7 seemed to prefer clonal expansion at the injection site rather than spread (Fig. 3D). Herein, we concluded that NSCLC cancer cells would not proliferate in the brain of zebrafish within four days after injection. So, the increase of NSCLC cell appeared in the brain of zebrafish were produced by metastasis from the circulation rather than local cell proliferation.

### Zebrafish NSCLC BM xenograft models discriminated the BM potential of different cell lines

**Fig. 4:**
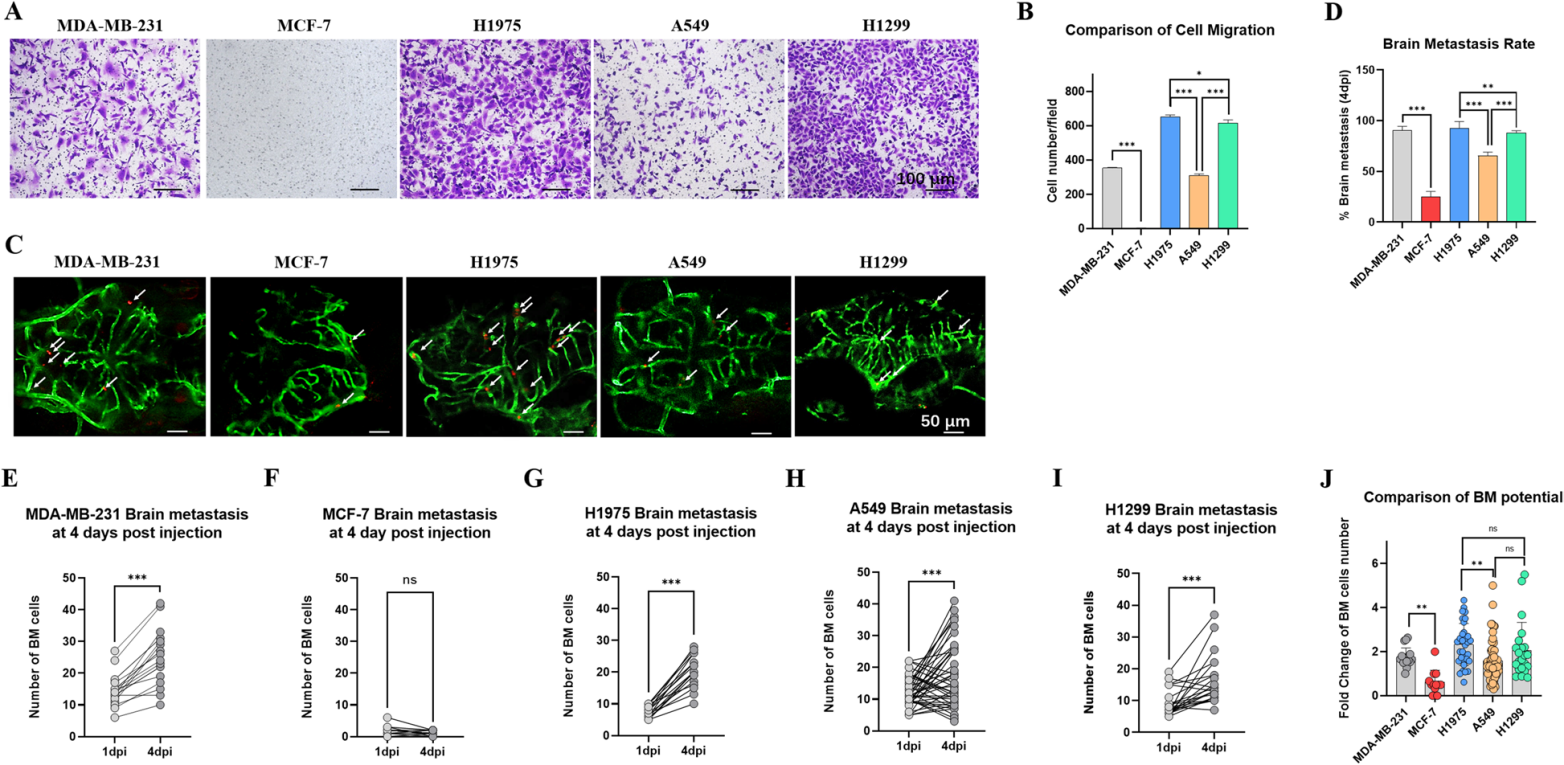
Zebrafish NSCLC BM xenograft models discriminated the BM potentials of different cell lines. (**A**) Transwell culture system was used to assess cell invasion ability *in vitro*, and five cancer cell lines were involved: MDA-MB-231, MCF-7, H1975, A549 and H1299. Invaded cells were stained with crystal violet (0.5 %) and imaged after 24 h incubation. (**B**) Quantification and comparison of invaded cells *in vitro*. Colonies were quantified using Image Pro Plus. (**C**) Five cancer cell lines were involved: MDA-MB-231, MCF-7, H1975, A549 and H1299. About 100 cells (red fluorescence) were injected into the PVs of Tg (*fli-1*: EGFP) zebrafish at 2 dpf, and the brain of zebrafish was imaged at 4 dpi. The white arrows indicated cancer cells in blood vessels of zebrafish brain. (**D**) Quantification of BM rate at 4 dpi. The number of BM cells in the same zebrafish at 4 dpi was divided by the number of BM cells at 1 dpi. The ratio of these two was named as BM potential. If the BM potential was greater than 1, it was considered that the zebrafish had brain metastasis. (**E**-**I**) Quantification of BM cells at 1 dpi and 4 dpi. The two dots connected by a straight line represented the number of BM cells of the same zebrafish at 1 dpi and 4 dpi. (**J**) Quantification and comparison of cell BM potential. The BM potential was determined by dividing the number of BM cells in the same zebrafish at 4 dpi by the number of BM cells at 1 dpi. (ns) indicated statistical insignificance, (*) indicated statistical significance P < 0.05, (**) P < 0.01 and (***) P < 0.001

Transwell culture system was used to assess cell invasion ability *in vitro*. Images and quantification indicated that MDA-MB-231, H1975, A549 and H1299 had strong invasion ability *in vitro*, and MCF-7 almost had no invasion ability (Fig. 4 A and B). MDA-MB-231 showed stronger invasion ability than MCF-7 even though they are both breast cancer cells, which was consistent with the previous study [33]. H1975 showed slightly stronger invasion ability than H1299. H1975 and H1299 both showed stronger invasion ability than A549 *in vitro*.

The *in vivo* BM abilities of the five cancer cell lines were assessed in zebrafish model. About 100 cells (red fluorescence) were injected into the PVs of Tg (*fli-1*: EGFP) zebrafish at 2 dpf. The brain of zebrafish was imaged at 4 dpi (Fig. 4 C) and the BM cells were quantified at 1 dpi and 4 dpi (Fig. 4E-I). In the same zebrafish, the number of BM cells at 4 dpi was divided by the number of BM cells at 1 dpi. The ratio of these two was named as BM potential. If the BM potential was greater than 1, it was considered that the zebrafish had brain metastasis. According to this statistical method, we determined the BM rate and BM potential of each cell line. H1975 showed higher BM rate and BM potential than A549 at 4 dpi (Fig. 4D and J), consistent with results *in vitro* (Fig. 4B). From the quantification of BM cells, almost all zebrafish injected with H1975 were individuals with increased number of BM cells (Fig. 4G), similar to the positive control (MDA-MB-231) (Fig. 4E). Despite H1299 showed higher BM rate than A549, it did not show higher BM potential than A549. According to the quantification of BM cells, we found that there were individuals with decreased number of BM cells in the zebrafish injected with H1299 (Fig. 4I), and this situation also appeared in the zebrafish injected with A549 (Fig. 4 H) or MCF-7 (Fig. 4 F). The difference between the zebrafish injected with MCF-7 and A549 was that almost all individuals with MCF-7 had reduced number of BM cells, while only part of individuals with A549 had reduced number of BM cells. We believed that compared to MCF-7, A549 had higher BM potential *in vivo*. Therefore, we concluded that our zebrafish BM models could discriminated the BM potential of different NSCLC cell lines.

### Zebrafish NSCLC BM xenograft models simultaneously discriminated the BM potential of different cell lines

**Fig. 5:**
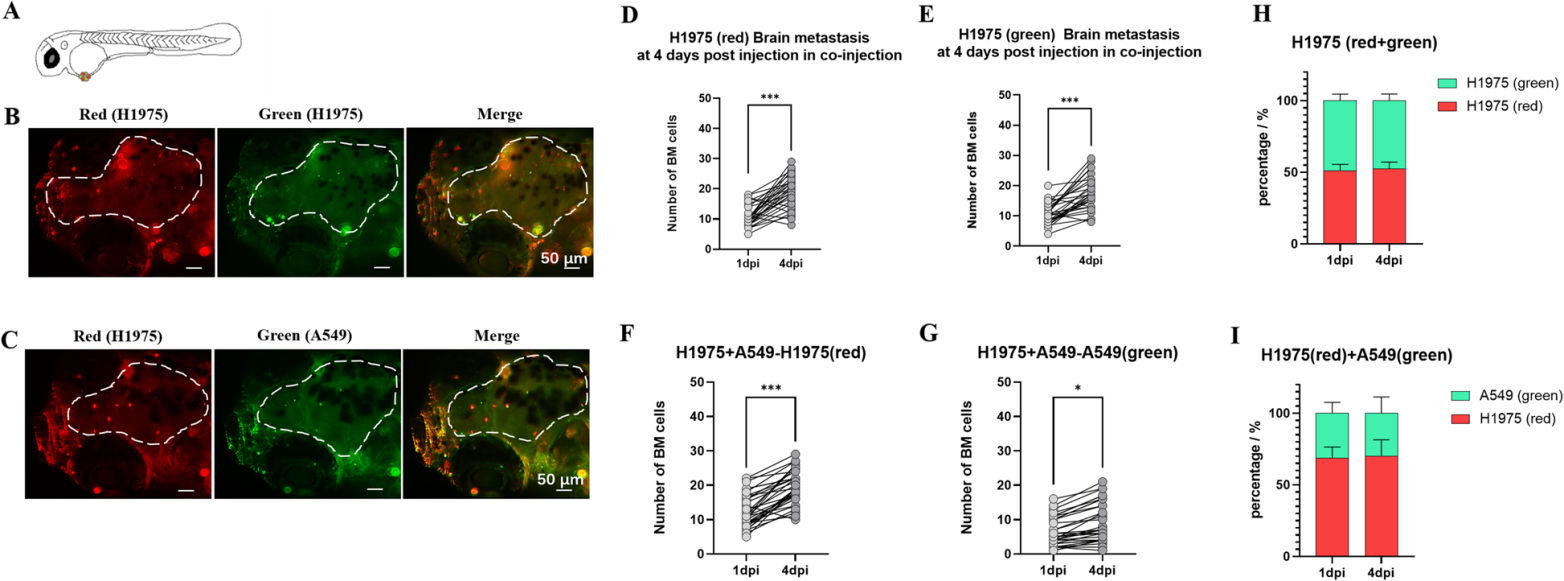
Zebrafish NSCLC BM xenograft models simultaneously discriminated the BM potentials of different cell lines. (**A**) The schematic diagram showed zebrafish embryos at 2 dpf. The red dots and green dots indicated cancer cells labeled with two different dyes were co-injected into the PVs of zebrafish. (**B**) About 50 H1975 cells (red fluorescence) and 50 H1975 cells (green fluorescence) were co-injected into the PVs of wild zebrafish at 2 dpf, and the brain of zebrafish was imaged at 4 dpi. The white long dashed line indicated the brain of zebrafish. (**C**) About 50 H1975 cells (red fluorescence) and 50 A549 cells (green fluorescence) were co-injected into the PVs of wild zebrafish at 2 dpf, and the brain of zebrafish was imaged at 4 dpi. (**D**-**G**) Quantification of BM cells in co-injection at 1 dpi and 4 dpi. The two dots connected by a straight line represented the number of BM cells of the same zebrafish at 1 dpi and 4 dpi. (**H** and **I**) The percentage of cells with two different dyes in the brain of zebrafish. The percentage of the cell line (red/green fluorescence) at 1 dpi was determined by dividing the number of BM cells (red/green fluorescence) by the total number of BM cells at 1 dpi. (ns) indicated statistical insignificance, (*) indicated statistical significance P < 0.05, (**) P < 0.01 and (***) P < 0.001

Since zebrafish BM model could distinguished the BM potential of different NSCLC cell lines, we would like to know whether this difference of BM potential could be displayed at the same time. As the schematic diagram shown, two NSCLC cell lines labeled with different fluorescent dyes (Fig. 5 A) were co-injected into the PVs of zebrafish. In order to eliminate the influence of different dyes on the BM potential of cell lines, H1975 cells were labeled with CM-DiI (red fluorescence) or CM-DiO (green fluorescence) separately, then mixed in equal proportions and co-injected (about 100 cells in sum) to the PVs of wild zebrafish at 2 dpf. The brain of zebrafish was imaged at 4 dpi (Fig. 5B) and the number of BM cells was quantified at 1 dpi and 4 dpi (Fig. 5D and E). The percentage of H1975 cells labeled in red at 4 dpi was determined by dividing the number of its BM cells by the total number of BM cells at 4 dpi. As shown in Fig. 5 H, the percentage of H1975 cells labeled in red was almost the same as the percentage of H1975 cells labeled in green at 4 dpi, which meant the different dyes had no effect on the BM potential of cell lines.

H1975 cells labeled in red and A549 cells labeled in green were co-injected (about 100 cells) into the PVs of zebrafish at 2 dpf. The brain of zebrafish was imaged at 4 dpi (Fig. 5 C) and the number of BM cells was quantified at 1 dpi and 4 dpi (Fig. 5 F and G). H1975 was considered to have higher BM potential than A549 in above study. When they were co-injected into the PVs, H1975 displayed its higher BM potential than A549, with the decreased BM potential of A549 (Fig. 4 H and Fig. 5G). The percentage of H1975 cells was also higher than that of A549 (Fig. 5I). It was noteworthy that the BM potential of H1975 was also lower than that in above studies (Figs. 4G and 5 F). We speculated that the co-existence of A549 may occupied the metastatic space of H1975 and vice versa.

### Expression of microRNA-330-3p (miR-330-3p) affected the NSCLC BM potential in zebrafish xenograft model

MiRNAs are known to play an important role in the development of many cancers. Recent studies have shown that miR-330-3p contributes to the occurrence of tumors in glioblastoma, colorectal cancer and esophageal cancer [34–36]. Elevated miR-330-3p expression promotes the proliferation, survival, migration and invasion of cancer cells *in vitro*, and tumor formation in nude mice [35]. miR-330-3p has also been shown to promote NSCLC invasion and metastasis, and may be a useful biomarker for identifying NSCLC with brain metastasis potential [36, 37]. Therefore, miR-330-3p manipulation was used to verify the zebrafish BM model.

**Fig. 6:**
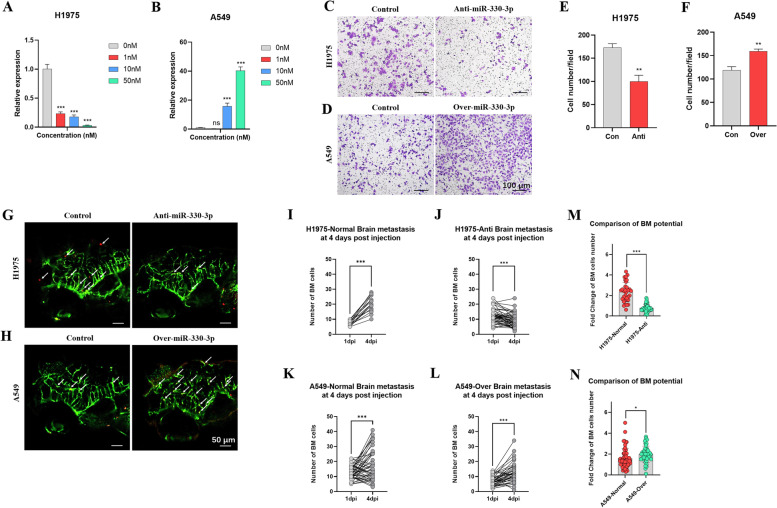
Expression of microRNA-330-3p (miR-330-3p) affected the NSCLC BM potential in zebrafish xenograft models. (**A** and **B**) Quantification of the miR-330-3p expression *in vitro*. The appropriate transfection concentration was determined between 1 nM and 50 nM, according to the instructions. (**C** and **D**) The expression of miR-330-3p in H1975 cell line was knocked down by anti-miR-330-3p inhibitor and the expression of miR-330-3p in A549 cell line was overexpressed by over-miR-330-3p mimics. Transwell culture system was used to evaluate the effectiveness of transfection *in vitro*. Invaded cells were stained with crystal violet (0.5 %) and imaged after 24 h incubation. (**E** and **F**) Quantification of invaded cells *in vitro*. Colonies were quantified using Image Pro Plus. (**G** and **H**) H1975, H1975 with under-expressed miR-330-3p, A549 and A549 with over-expressed miR-330-3p were involved. About 100 cells (red fluorescence) were injected into the PVs of Tg (*fli-1*: EGFP) zebrafish at 2 dpf to evaluate the effectiveness of transfection *in vivo*, and the brain of zebrafish was imaged at 4 dpi. The white arrows indicated cancer cells in blood vessels of zebrafish brain. (**I**-**L**) Quantification of BM cells at 1 dpi and 4 dpi. The two dots connected by a straight line represented the number of BM cells of the same zebrafish at 1 dpi and 4 dpi. (**M** and **N**) Quantification and comparison of the BM potentials of the transfected cell lines and the original cell lines. (ns) indicated statistical insignificance, (*) indicated statistical significance P < 0.05, (**) P < 0.01 and (***) P < 0.001

The expression of miR-330-3p in H1975 cell line was knocked down by anti-miR-330-3p inhibitor and the expression of miR-330-3p in A549 cell line was overexpressed by over-miR-330-3p mimics. The appropriate transfection concentration was determined between 1 nM and 50 nM, according to the manufacturer’s instructions. The quantification of the miR-330-3p expression *in vitro* indicated 50 nM was the appropriate concentration (Fig. 6. A and B). Transwell culture system was used to evaluate the effectiveness of transfection *in vitro*. H1975 cell lines with knocked down expression of miR-330-3p showed weaker invasion ability than original H1975 cell lines (Fig. 6 C and E). A549 cell lines with over-expressed miR-330-3p showed stronger invasion ability than original A549 cell lines (Fig. 6D and F).

H1975, H1975 with under-expressed miR-330-3p, A549 and A549 with over-expressed miR-330-3p were involved in zebrafish study. About 100 cells were injected into the PVs of Tg (*fli-1*: EGFP) zebrafish at 2 dpf to evaluate the effectiveness of transfection *in vivo*. The brain of zebrafish was imaged at 4 dpi (Fig. 6G and H) and the number of BM cells was quantified at 1 dpi and 4 dpi (Fig. 6I-L). Data showed significantly decreased BM potential of H1975 cell line with under-expressed miR-330-3p, and A549 with over-expressed miR-330-3p showed higher BM potential than the original A549 cell line (Fig. 6 M and N).

### Zebrafish NSCLC BM xenograft model discriminated different chemosensitivities within 4 days

**Fig. 7:**
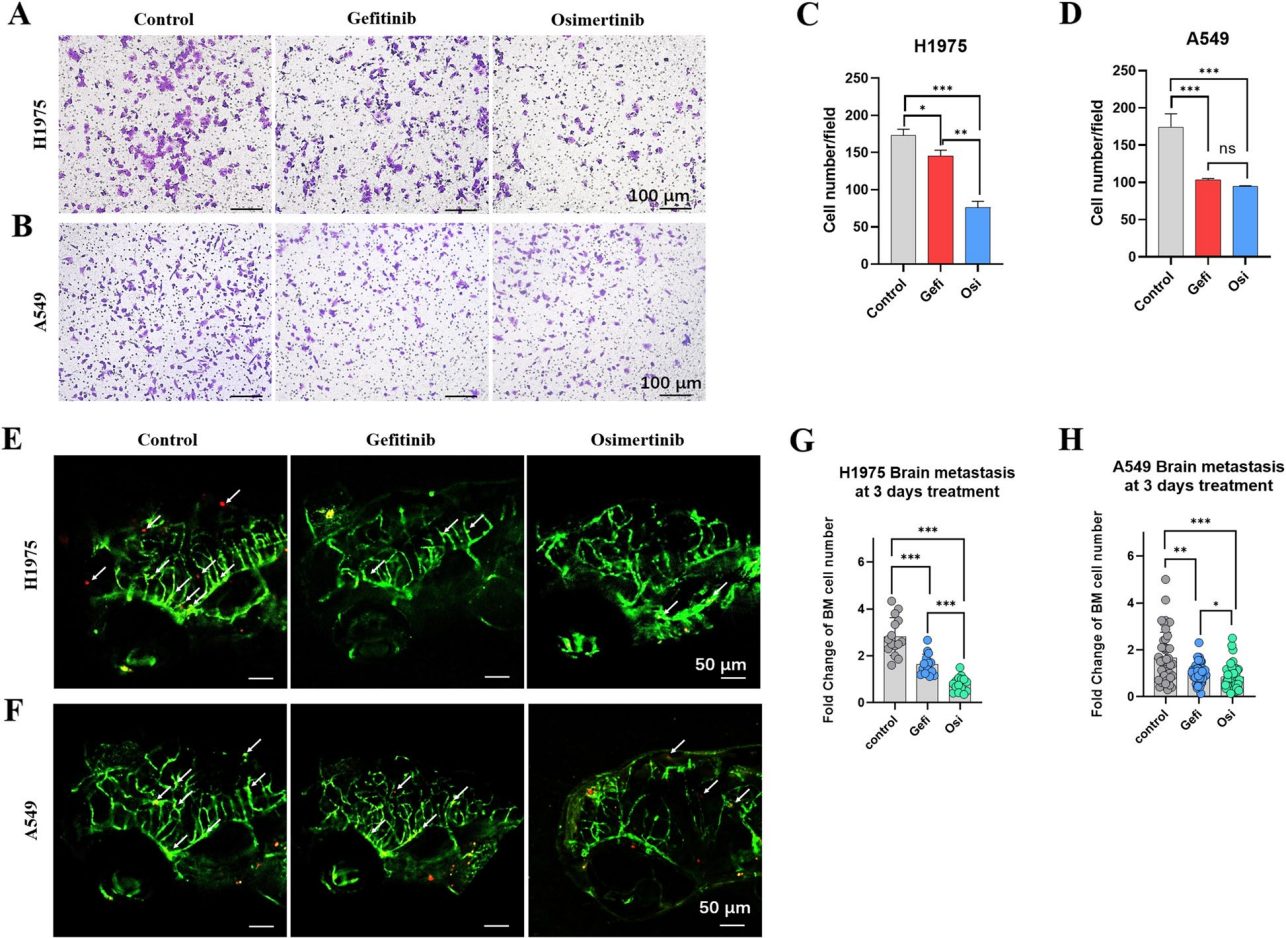
Zebrafish NSCLC BM xenograft models discriminated different chemosensitivities within 4 days. (**A** and **B**) NSCLC cell lines H1975 and A549 were involved. Transwell culture system was used to evaluate the effectiveness of osimertinib (1 µM) and gefitinib (2 µM) against cell invasion *in vitro*. Invaded cells were stained with crystal violet (0.5 %) and imaged after 24 h incubation. (**C** and **D**) Quantification of invaded cells in vitro. Colonies were quantified using Image Pro Plus. (**E** and **F**) NSCLC cell lines H1975 and A549 were involved. Tg (*fli-1*: EGFP) zebrafish embryos were injected about 100 cells (red fluorescence) into the PVs at 2 dpf and administered with osimertinib (1 µM) and gefitinib (13 µM) by intracardiac injection. (**G** and **H**) Quantification of cell brain metastasis at 4 dpi. Fold change of BM cell number was determined by dividing the number of BM cells in the same zebrafish at 4 dpi by the number of BM cells at 1 dpi. (ns) indicated statistical insignificance, (*) indicated statistical significance P < 0.05, (**) P < 0.01 and (***) P < 0.001

Osimertinib, one of the third-generation epidermal growth factor receptor-tyrosine kinase inhibitors (EGFR-TKIs), had an established efficacy and safety profile as first- and second-line therapy in NSCLC, it can penetrate BBB and showing better efficacy than gefitinib in BM patients. Transwell culture system was used to evaluate the effectiveness of osimertinib (1 µM) and gefitinib (2 µM) against cell invasion *in vitro* (Fig. 7 A and B). Despite osimertinib showed stronger inhibition of cell invasion than gefitinib on H1975, there was no significant difference between the two on A549 (Fig. 7 C and D).

Human NSCLC cell lines H1975 and A549 were involved to evaluate the effectiveness of osimertinib (1 µM) and gefitinib (13 µM) *in vivo*. About 100 cells were injected into the PVs of Tg (*fli-1*: EGFP) zebrafish at 2 dpf and embryos with brain metastasis were administered with osimertinib and gefitinib by intracardiac injection at 1 dpi. The brain of zebrafish was imaged at 4 dpi (Fig. 7E and F) and the number of BM cells was quantified at 1 dpi and 4 dpi (Fig. 7G and H). Consistent with results *in vitro*, osimertinib showed stronger inhibition of BM potential than gefitinib on H1975 *in vivo* (Fig. 7 C and G). Unlike *in vitro*, osimertinib showed stronger inhibition of BM potential on A549 than gefitinib *in vivo* (Fig. 7D and H).

## Discussion

The increased incidence of BM in NSCLC patients and dismal prognosis had promoted the urgency to understand the pathophysiology of BM and to test effective therapeutic strategies for these patients [28]. Although many advances had been made in the treatment of NSCLC patients, the prognosis of BM patients was still dismal. Targeted drugs used for the treatment of NSCLC BM were limited by the BBB from entering the brain parenchyma, thereby reducing the efficacy [13]. Drug resistance was also a common problem in NSCLC BM, especially the lack of the fourth-generation tyrosine kinase inhibitors after osimertinib resistance [38]. Besides, the time left for the treatment of brain metastases from lung cancer was short, whose median survival time was usually 3 to 8 months [5, 13]. Thus, in the current scenario, it is necessary to conduct more mechanism-associated research and verification of new therapy strategies for NSCLC BM.

Animal models were gradually becoming important auxiliary tools for cancer research. The current mainstream animal model for cancer research is the mouse model. However, mouse models are not practical for clinical advice due to the time it takes to implant tumors and expand colonies, and the costs they entailed [21]. After all, BM is a common metastasis of advanced cancer, while it often took one month or more for mouse models to reach advanced cancer [39]. Here, we had taken an intermediate approach—a fast and reliable zebrafish NSCLC BM model.

Our model could well simulate the association between BBB and NSCLC BM. Zebrafish has a BBB that is structurally and functionally similar to that of mammals [29]. The acquisition of BBB properties occurs in brain endothelial cells during angiogenesis starting at 30 hpf, and the BBB continues to mature, with increasing restriction of lower molecular weight substances with age, assessed up to 10 dpf [40, 41]. In our study, the BM potentials of H1975, A549 and H1299 injected at 2 dpf were significantly higher than that injected at 4 dpf, which meant the BM potential of tumor cells could be weakened by the development of BBB. Our data also showed that BBB with more complete function and structure had a higher barrier effect on cancer cells, but it could not completely intercept the brain invasion of lung cancer cells (Fig. 1 F-K). Although zebrafish BBB at 5 dpf has more clinical value than the zebrafish BBB at 2 dpf, we still chose 2 dpf as the injection time for some reasons. First, from 2 dpf to 6 dpf, the main innate immune cells in zebrafish are macrophages and neutrophils, which might play an important role in tumor clearance. Rita group has demonstrated that the recruitment of macrophages and neutrophils in zebrafish resulted in the poor implantation effect of colorectal cancer cell line SW480 [42]. Most studies on T cell development in zebrafish are carried out after 5 dpf, because the lymphoid progenitor cells colonize the thymus at about 5 dpf and leave the thymus at about 6 dpf to 7 dpf to enter the peripheral circulation [43]. Similar to macrophages and neutrophils, T cells are also the main effector cells of the immune system that directly target xenografted cancer cells [44]. In our experiments, we found that when 100 cancer cells were transplanted into the zebrafish yolk sac at 2 dpf, the growth of the cells slowed down after 5 dpf (3 dpi) (Figure S1A). The same situation occurred when 100 cancer cells were injected into the PVs of the 2 dpf zebrafish. The increase of the BM was also slowed down after 5 dpf (3 dpi) (Figure S1B). We speculated that it might be due to the development of the innate immune system after 5 dpf, and it begins to reject the xenografted cancer cells. Second, the PVs is a cavity around the zebrafish yolk sac without organs or blood vessels. The upper part of the PVs is connected to the man venous tube (usually called the Cuvier tube) of zebrafish, and the front part is connected to the pericardium (the aortic blood vessels in the pericardium) [45]. Therefore, the PVs is connected to the zebrafish vasculature, which is conductive to the metastasis of cancer cells. Through injection into the PVs, the cancer cells could not only metastasize to the brain but also to the tail (Fig.S2). Many cancer metastasis studies based on the zebrafish model have used the PVs as the injection site [46, 47]. We observed that the PVs of zebrafish almost completely disappeared at 5 dpf, which would affect the accuracy of the microinjection site. Third, it was reported that the BBB may be damaged when brain metastases occur [48]. It was reported that when metastatic tumors grow beyond 1-2 mm within the patient brain parenchyma, the BBB became structurally and functionally compromised. Perturbation of the BBB may lead to an increase in permeability and an edematous response [49, 50]. Fourth, it will be more statistical reliable when the BM cells reach more than 10, which hardly occurred when the cells were injected after 4 dpf.

After confirming that our model could develop NSCLC BM, we conducted related verification of NSCLC BM mechanisms and drug sensitivity. After all, the purpose of establishing the zebrafish BM models was to provide assistance for the study of NSCLC BM mechanisms and the verification of new therapy strategies. The difficulty in the treatment of NSCLC BM was the unclear mechanism, and the inaccurate medication caused by multiple targets and individual differences [28]. We verified the reported effects of miR-330-3p on BM of NSCLC [37], and the application of EGFR-targeted drugs—gefitinib and osimertinib in the treatment of NSCLC BM [51, 52]. When verifying the effects of miR-330-3p on the BM potentials in NSCLC cell lines, compared with the reduction of the BM potential in the H1975 cell line with under-expressed miR-330-3p, there was a little increase of the BM potential in the A549 cell line with over-expression of miR-330-3p *in vivo* (Fig. 6G-N). According to clinical reports, the expression of miR-330-3p had a slight increase in NSCLC BM+ patients, while the expression of miR-330-3p was significantly reduced in NSCLC BM- patients, which meant miR-330-3p could be a BM- marker [53].

In most of the zebrafish xenograft models, drug exposures were generally transdermal absorption (soaking) [20, 21]. We initially tried to deliver drugs by transdermal absorption, but this administration method did not distinguish the effects of osimertinib and gefitinib on NSCLC BM cells (data not shown). We speculated that transdermal absorption allowed gefitinib to bypass the existence of BBB and directly acted on cancer cells that metastasized to the brain of zebrafish. From this respect, anti-BM drugs should have the ability to infiltrate BBB. In our zebrafish BM model, it needs to wait for a fully functional BBB before drug injection. We designed experiments in which the drug treatments were from 3 dpf to 6 dpf and 5 dpf to 8 dpf to make a direct comparison (Fig. S3). Results showed that the BM cells of all three cell lines (H1975, A549, and H1299) decreased in the control groups in 5-8 dpf treatment protocol compared to those in 3-6 dpf treatment protocol. Drugs administrated at 5 dpf still have significant inhibitory effects on the BM of cancer cells, similar to the effect of corresponding drugs administrated at 3 dpf, except that osimertinib showed stronger inhibition effect than gefitinib in H1975 cells when the drug exposure time was 3-6 dpf, but this difference did not reproduce in H1975 cells when the drug exposure time was 5-8 dpf. We finally chose 3 dpf as the initiation of drug treatment according to the following four aspects. First, at 8 dpf, the melanin in zebrafish brain hindered the observation of BM cells despite the pretreatment of PTU. Higher concentrations of PTU will cause abnormal development of the juvenile zebrafish. Second, cancer cells were fluorescently labelled with CM-DiI, this dye (and most of the fluorescent dyes) will fade gradually with the cell division. At 8 dpf (6 dpi), cells signal became weak, which led to inaccurate observation and BM cell quantification. Third, zebrafish immune cells might participate in the tumor cell clearance at 5-8 dpf. This may be also one of the reasons why the drug sensitivity difference did not reproduce in H1975 cells when exposure delayed to 5-8 dpf (Fig.S3A and A’). Fourth, we have mentioned that the expression of *mfsd2aa* mark the beginning of BBB maturation, the first expression of *mfsd2ab* mark the mature functional BBB in the midbrain of zebrafish. The BBB of zebrafish may begin to functional develop at 3 dpf, basically developed at 4 dpf, and fully developed at 5 dpf. Specifically, the midbrain of zebrafish (the main site of BM cells and also the main site of observation) at 3 dpf already had relatively complete functional micro vessels, but the micro vessels of the hindbrain were still developing [29]. Therefore, we delivered the drugs by intracardiac injection at 3 dpf (1 dpi) in which the drugs were directly delivered into the blood stream, they had to go through BBB to reach the midbrain and finally acted on the cancer cells that metastasized to the brain.

In this study, we found that the NSCLC cell line H1975 was more sensitive to osimertinib than gefitinib both *in vivo* and *in vitro* (Fig. 7 C and G). This was in line with clinical reports that in NSCLC BM patients with EGFR mutations, osimertinib had shown greater efficacy than other EGFR-TKIs due to its better BBB permeability and better efficiency [52]. However, a similar situation did not occur in A549. Although A549 was sensitive to both osimertinib and gefitinib, it could not clearly reflect the difference in efficacy between osimertinib and gefitinib like H1975 *in vivo* and *in vitro* (Fig. 7D and H). In other words, compared with H1975, the better efficacy and BBB permeability of osimertinib over gefitinib were not reflected in A549. Osimertinib was a targeted inhibitor of T790, while H1975 had T790M and L858R mutations and A549 did not. The absence of this exact target may make osimertinib more effective in H1975 than in A549. Gefitinib targeted EGFR 19/21 exon mutations. EGFR 19/21 exon mutations were often accompanied by an increase in EGFR expression [54], and the expression of EGFR in A549 was higher than that in H1975, which may promote gefitinib to have a stronger drug effect in A549 than in H1975. This result may cause the difference in efficacy between gefitinib and osimertinib, which could only be reflected in H1975 but not in A549. All in all, it proved that our BM models could distinguish the chemical sensitivity of NSCLC BM to targeted drugs. In addition, this difference was manifested very quickly—only three days after administration, which met the clinical needs of rapid.

Of course, the biggest disadvantage of zebrafish BM models was that the tumor could not exist for a long time in brain of zebrafish. Zebrafish embryos had a complete immune system at one month post fertilization [55]. The inability of bearing tumors in long time in zebrafish meant the inability of the study of tumor evolution, emergence of resistance clones, and overall progression of disease in zebrafish xenograft model [21]. At this point, a mouse model was needed to complement it. The zebrafish NSCLC BM models could be used for rapid screening or rapid verification of targets, while the mouse BM model could provide solid BM tumors to study tumor progression and recurrence.

With the increasing research using zebrafish as a model for patient-derived xenograft (zPDX) [20, 21], zebrafish has proved to be a rapid and accurate model that can be used to display a variety of biological tumor characteristics and evaluate tumor response to treatment. This model can be used not only for basic research, but also for clinical precision medicine [21]. It is important to use patient-derived primary cultures in order to better understand the biology and malignant processes of NSCLC. Although 3D cultures and bioreactors perform well *in vitro*, verification based on *in vivo* models is indispensable for patient-derived primary cultures. The choice of model takes on an essential importance to guarantee the greatest advantages from primary cultures [59]. Our experimental results showed that the zebrafish model can well retain and display the brain metastasis characteristics of the NSCLC cell line, which lays the foundation for the application of the zebrafish model to primary cultures. We hope we could further use our zebrafish BM model for the patient-derived xenograft to validate its potential application in the clinical practice.

## Conclusions

In summary, we established the zebrafish NSCLC BM models for the first time and showed that the zebrafish NSCLC BM models were fast and reliable models sensitive to cell heterogeneity. We performed confirmatory experiments with NSCLC cell lines, which provided a new platform for the research of NSCLC BM. We may even provide new possibilities for rapid screening of personalized NSCLC BM treatment in clinic.

## Supplementary Information

**Additional file 1: Fig. S1.** Proliferation and brain metastasis of H1975. (A) H1975 cells were microinjected in yolk sac of zebrafish embryo at 2 dpf. Cell proliferations were assessed from 1 dpi to 4 dpi. (B) H1975 cells were microinjected in PVs of zebrafish embryo at 2 dpf. Cell brain metastasis were assessed from 1 dpi to 4 dpi. Significance was considered when P values were lower than 0.05. (ns) indicated statistical insignificance, (*) indicated statistical significance P < 0.05, (**) P < 0.01 and (***) P < 0.001. dpf: days post fertilization, dpi: days post injection.

**Additional file 2: Fig. S2.** Comparison of the anti-metastasis effects of gefitinib and osimertinib administrated at 3 dpf and 5 dpf on zebrafish model. About 100 cells of H1975, A549 and H1299 were injected into the PVs of Tg (fli-1: EGFP) zebrafish embryos at 2 dpf. Osimertinib (1 μM) and gefitinib (13 μM) were administrated by intracardiac injection at 3 dpf (A, B and C) or 5 dpf (A’, B’ and C’). BM cells were quantified after three days exposure. Fold change of BM cell number were determined by dividing the number of BM cells at exposure end by the number of BM cells at exposure initiation. (ns) indicated statistical insignificance, (*) indicated statistical significance P < 0.05, (**) P < 0.01 and (***) P < 0.001.

**Additional file 3: Fig. S3.** Brain and tail metastasis of H1975 cell at 1 day post injection. About 100 H1975 cells (labeled with red fluorescent dye) were injected into the PVs of 2 dpf Tg (*fli-1*: EGFP) zebrafish, at 3 dpf (1 dpi), metastasis to the tail and brain could be clearly seen. The white arrow points to the cancer cell injection site. The white dashed box showed the brain metastasis and tail metastasis.

## Data Availability

The datasets used and/or analysed during the current study are available from the corresponding author on reasonable request.

## Abbreviations

NSCLC: Non-small cell lung cancer
BM: Brain metastasis
BBB: Blood-brain barrier
ALK: Anaplastic lymphoma kinase
Osi: Osimertinib
Gefi: Gefitinib
FBS: Fetal bovine serum
PBS: Phosphate buffer saline
RPMI 1640: Roswell Park Memorial Institute basal medium 1640
PTU: N-phenylthiourea
dpf: days post-fertilization
IACUC: Institutional Animal Care and Use Committee
dpi: days post injection
qPCR: Quantitative Real-time PCR
HE: Hematoxylin-eosin
PVs: perivitelline space
miR-330-3p: microRNA-330-3p
EGFR-TKI: epidermal growth factor receptor-tyrosine kinase inhibitor
