# Supplementary figures and images for: Zebrafish xenograft model for studying mechanism and treatment of non-small cell lung cancer brain metastasis
Source: J Exp Clin Cancer Res. 2021 Nov 20;40:371. doi: 10.1186/s13046-021-02173-5 (PMC8605597; doi:10.1186/s13046-021-02173-5)

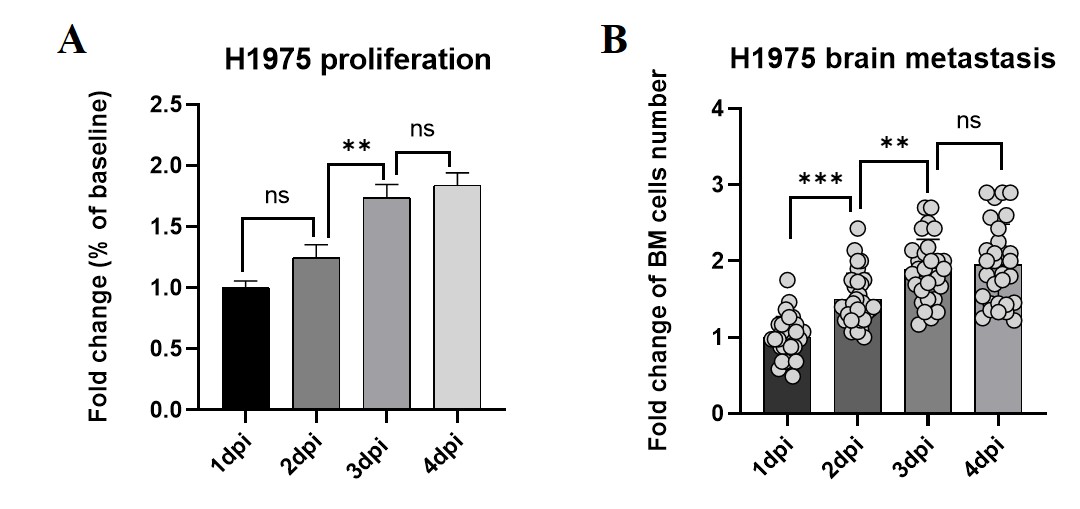

Supplement: Supplementary file 1 — Additional file 1: Fig. S1. Proliferation and brain metastasis of H1975. (A) H1975 cells were microinjected in yolk sac of zebrafish embryo at 2 dpf. Cell proliferations were assessed from 1 dpi to 4 dpi. (B) H1975 cells were microinjected in PVs of zebrafish embryo at 2 dpf. Cell brain metastasis were assessed from 1 dpi to 4 dpi. Significance was considered when P values were lower than 0.05. (ns) indicated statistical insignificance, (*) indicated statistical significance P < 0.05, (**) P < 0.01 and (***) P < 0.001. dpf: days post fertilization, dpi: days post injection. [file 13046_2021_2173_MOESM1_ESM.jpg]

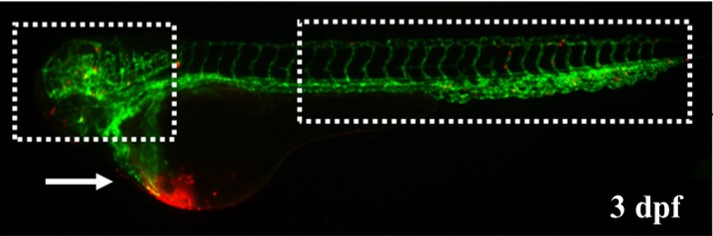

Supplement: Supplementary file 2 — Additional file 2: Fig. S2. Comparison of the anti-metastasis effects of gefitinib and osimertinib administrated at 3 dpf and 5 dpf on zebrafish model. About 100 cells of H1975, A549 and H1299 were injected into the PVs of Tg (fli-1: EGFP) zebrafish embryos at 2 dpf. Osimertinib (1 μM) and gefitinib (13 μM) were administrated by intracardiac injection at 3 dpf (A, B and C) or 5 dpf (A’, B’ and C’). BM cells were quantified after three days exposure. Fold change of BM cell number were determined by dividing the number of BM cells at exposure end by the number of BM cells at exposure initiation. (ns) indicated statistical insignificance, (*) indicated statistical significance P < 0.05, (**) P < 0.01 and (***) P < 0.001. [file 13046_2021_2173_MOESM2_ESM.jpg]

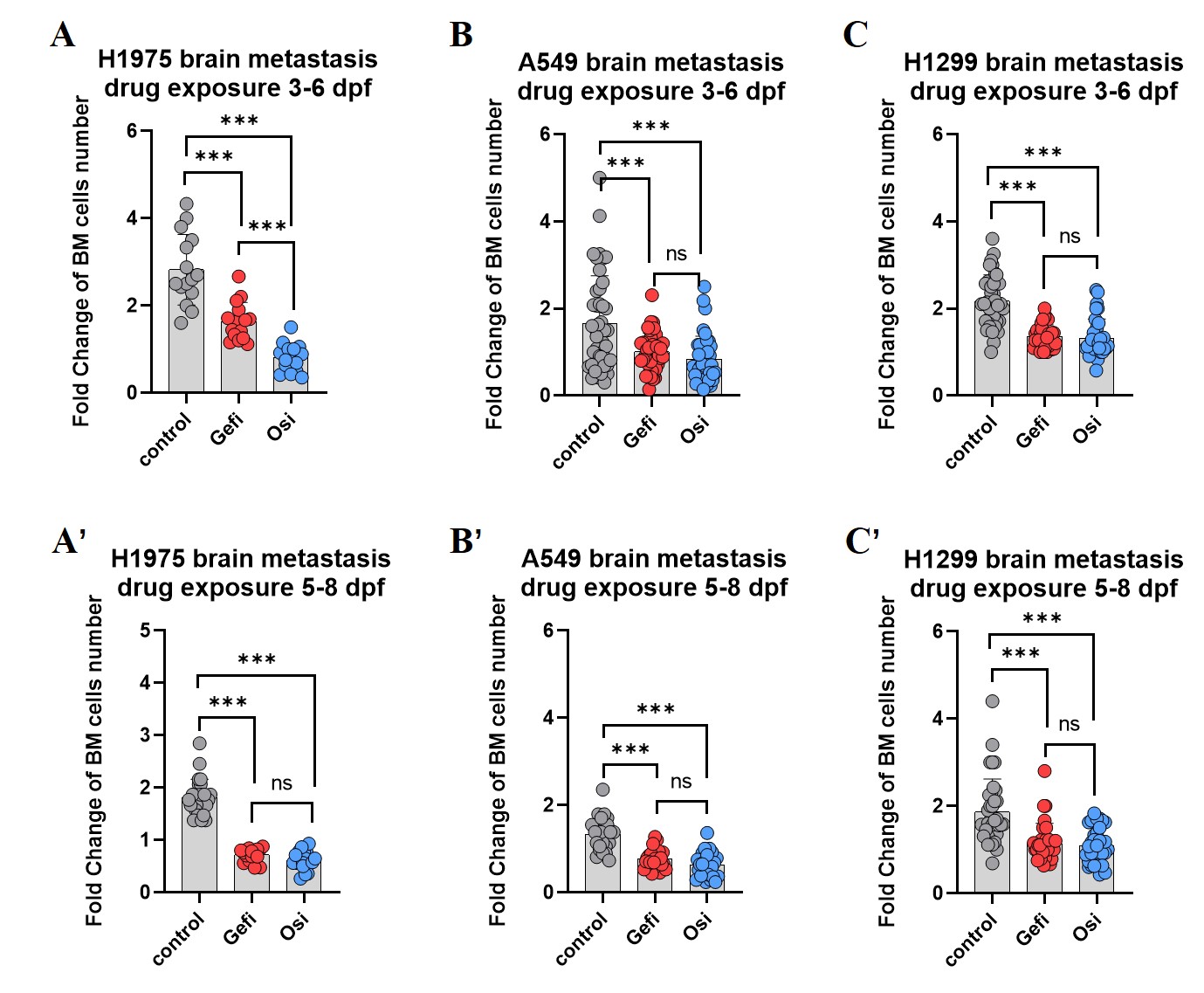

Supplement: Supplementary file 3 — Additional file 3: Fig. S3. Brain and tail metastasis of H1975 cell at 1 day post injection. About 100 H1975 cells (labeled with red fluorescent dye) were injected into the PVs of 2 dpf Tg (fli-1: EGFP) zebrafish, at 3 dpf (1 dpi), metastasis to the tail and brain could be clearly seen. The white arrow points to the cancer cell injection site. The white dashed box showed the brain metastasis and tail metastasis. [file 13046_2021_2173_MOESM3_ESM.jpg]
